# Human Neurospheroid Arrays for *In Vitro* Studies of Alzheimer’s Disease

**DOI:** 10.1038/s41598-018-20436-8

**Published:** 2018-02-05

**Authors:** Mehdi Jorfi, Carla D’Avanzo, Rudolph E. Tanzi, Doo Yeon Kim, Daniel Irimia

**Affiliations:** 1 Center for Engineering in Medicine, Department of Surgery, Massachusetts General Hospital, Harvard Medical School, Charlestown, Massachusetts 02129 USA; 2 Genetics and Aging Research Unit, MassGeneral Institute for Neurodegenerative Disease, Massachusetts General Hospital, Harvard Medical School, Charlestown, Massachusetts 02129 USA

## Abstract

Neurospheroids are commonly used for *in vitro* disease modeling and drug screening. However, the heterogeneity in size of the neurospheroids mixtures available through current methods limits their utility when employed for basic mechanistic studies of neurodegenerative diseases or screening for new interventions. Here, we generate neurospheroids from immortalized neural progenitor cells and human induced pluripotent stem cells that are uniform in size, into large-scale arrays. In proof of concept experiments, we validate the neurospheroids array as a sensitive and robust tool for screening compounds over extended time. We show that when suspended in three-dimensional extracellular matrix up to several weeks, the stem cell-derived neurospheroids display extensive neurite outgrowth and extend thick bundles of dendrites outward. We also cultivate genetically-engineered stem cell-derived neurospheroids with familial Alzheimer’s disease mutations for eight weeks in our microarray system. Interestingly, we observed robust accumulation of amyloid-β and phosphorylated tau, key hallmarks of Alzheimer’s disease. Overall, our *in vitro* model for engineering neurospheroid arrays is a valuable tool for studying complex neurodegenerative diseases and accelerating drug discovery.

## Introduction

Alzheimer’s disease (AD) affects more than 46 million people worldwide, at a global cost of US $818 billion in 2015^1–3^. Early-onset familial Alzheimer’s disease (FAD) is the hereditary form of AD that appears at an early age and is caused by three known genes mutations: amyloid precursor protein (APP), presenilin 1 (PSEN1), and presenilin 2 (PSEN2)^4,5^. AD is characterized by two main pathological hallmarks including deposition of amyloid-β (Aβ) plaques, and accumulation of hyperphosphorylated tau (p-tau) protein in axons, dendrites, and cell bodies^4,6^. To date, despite significant progress in understanding the biology underlying the AD pathogenesis, no cure exists for this devastating neurodegenerative disease. This is mainly due to the complexity of the human brain physiology, the limited access to the human brain tissues, and the limitations of current *in vivo* and *in vitro* models to fully recapitulate the disease phenotype. Lengthy ‘preclinical’ or ‘prodromal’ phase of AD preceded the onset of cognitive impairment by nearly seven years, posing further challenges for clinical trials of new AD treatments and therapies^7^.

Animal studies have contributed immensely to our understanding of the human brain functions^8^. However, current animal models do not always fully recapitulate the human brain physiology. For example, mouse models with FAD mutations show toxic Aβ production, but they lack neurofibrillary tangles, another key hallmark of the AD in human^9,10^. It has also been very challenging to fully recreate the AD pathogenesis with induced pluripotent stem cell (iPSC) from FAD patients models, likely due to the presence of low level of toxic Aβ in the system^11–13^. These and other limitations of animal disease models has delayed the progress towards finding a therapeutic target for AD^14,15^. Better physiologically-relevant disease models are needed to closely recapitulate AD pathology *in vitro*^16^. For the past five years, an increasing number of reports described *in vitro* disease models that successfully captured various aspects of AD pathology using human neurons derived from iPSC^17–30^. While the AD-relevant phenotypes observed in iPSC-neurons belong to early stages of the disease, these iPSC models have enabled significant insights into AD molecular mechanisms.

Two-dimensional (2D) cell culture dishes have been used for over a century to study cell biology. Although current 2D cell culture systems offer low cost and simplified platforms for studying AD pathology *in vitro*, some important characteristics of the brain diseases pathology cannot be reconstructed in typical dishes. This is mainly due to the complexity of human brain physiology, and its unique features^31^. Recently, three-dimensional (3D) neural cell culture models including brain organoids and neurospheroids have emerged and demonstrated significant potential as *in vitro* platforms for studying human brain cell biology^32–37^. We previously showed that genetically-engineered human neural stem cells that overexpress genes with multiple FAD mutations, combined with 3D Matrigel condition could recapitulate robust AD pathogenesis including the deposition of Aβ plaques and tangles in an AD-relevant 3D neural cell culture^38,39^. The current neurospheroids models utilize cells isolated from primary rat cortex, maintained in culture for relatively short-term frame of one-two weeks and rely on self-organization of the cells into neural spheroids^40–44^. These models were leveraged to recapitulate the disease phenotype of AD, and glioblastoma, and could provide a reliable alternative to current animal models^45–47^. However, these neurospheroid platforms are difficult to manipulate experimentally and rely on mixtures of spheroids that are heterogeneous in size, which limits the precision of analysis. Neurospheroids models are also sensitive to small variations including cell culture procedure, cell density, passage, and medium composition. This leads to neurospheroids that are heterogeneous in biological properties, stage of differentiation, as well as cell composition^48,49^. Altogether, it is very challenging to reproduce and consolidate the data between different studies and even different batches^48^. To show the potential of this exciting 3D brain cell culture model, it is essential to engineer a physiologically-relevant platform capable of generating viable, uniform-sized neurospheroids derived from human stem cells. Although neurospheroids have been shown to be extremely useful to reproduce neurological disease phenotypes, no previous study had applied this system to model specific-disease-like phenotypes *in vitro* using homogeneous arrays of human neurospheroids derived from stem cells.

Here, we leverage new tools and stem cell engineering to generate large-scale arrays of physiologically-relevant neurospheroids from genetically-engineered human neural stem cells with FAD mutations or human iPSC-derived neural progenitor cells. With this approach, we succeeded in generating neurospheroids that extend thick dendrites outward and give rise to mature neurons over the course of two to eight weeks in 3D cell culture. We show that after eight weeks in culture, the 3D neurospheroids display two main pathological events of AD, namely the accumulation of Aβ plaques and phosphorylated tau. We also validate the neurospheroids array as a reliable and predictive test bed for high-throughput drug screening.

## Results

**Figure 1:**
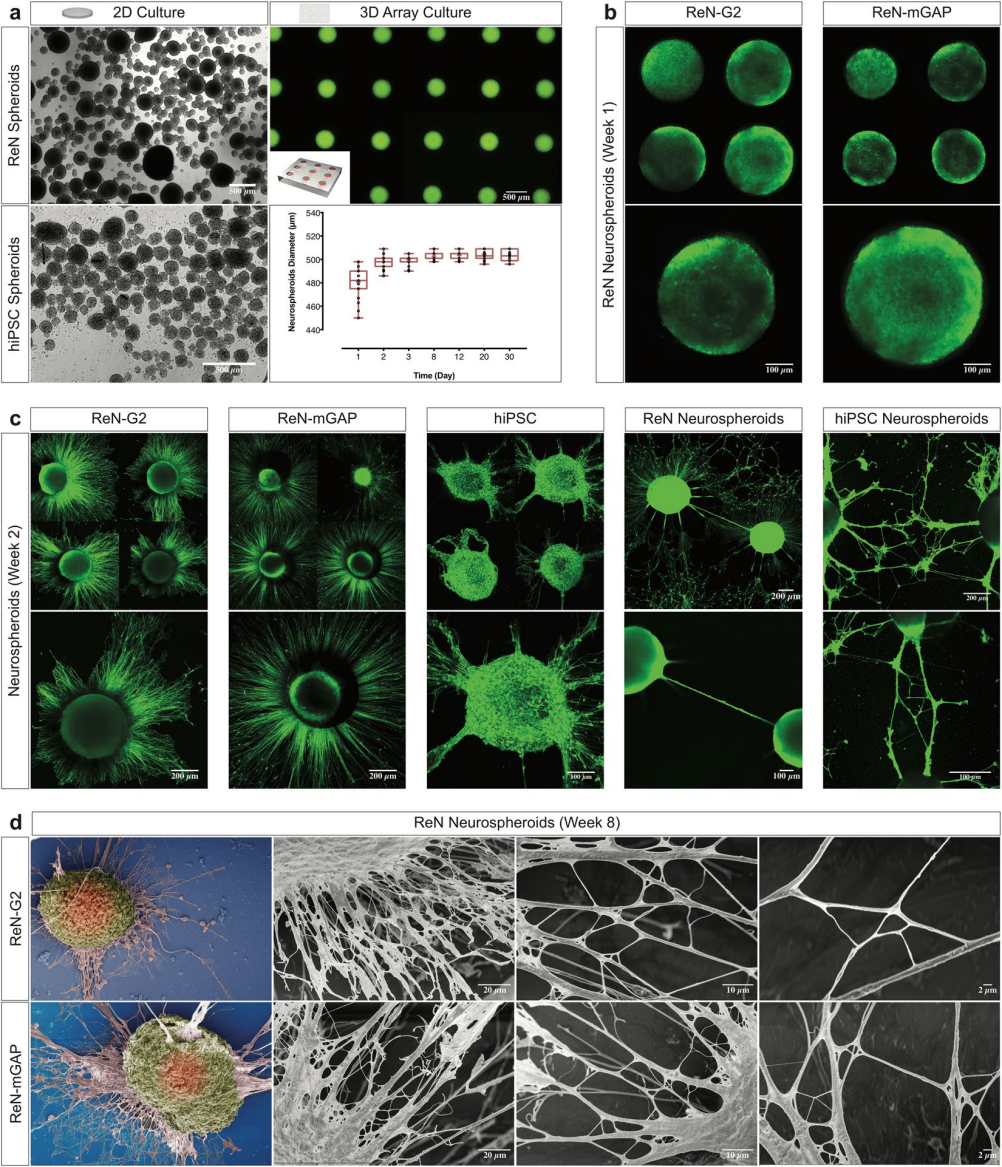
Generation and 3D differentiation of homogenously-sized human neurospheroids in a 3D array platform. (**a**) The left images show that ReN- and hiPSC-derived neurospheroids generated in 2D cell culture dishes are heterogenous in size (phase contrast). The right image shows homogenously-sized neurospheroids assembled in the 3D array platform (fluorescence of green fluorescent protein - GFP). The graph shows a quantification of ReN-derived neurospheroids diameter over the course of one month in the 3D array platform. (**b**) Images show expression of GFP in ReN-G2 (control) and ReN-mGAP-derived neurospheroids (FAD) generated in the 3D platform with Matrigel at 1-week differentiation. (**c**) Representative confocal images show 3D differentiation of ReN- and hiPSC-derived neurospheroids and extension of processes between adjacent neurospheroids at 2-week differentiation. (**d**) Scanning electron microscopy (SEM) images show bundles of neurites of ReN- and ReN-mGAP-derived neurospheroids at 8-week differentiation in the 3D array platform.

We generated stem-cell-derived neurospheroids from genetically-engineered ReNcell VM human neural stem (ReN) cells^38,39^ and human iPSC-derived neural progenitor cells (hiPSC) using microfabricated arrays of microwells. Unlike the neurospheroids cultured using traditional methods^45^, which are heterogeneous in size (100–800 µm diameter, Fig. 1a), neurospheroids formed by plating ReN and hiPSC cells in the microwells array (Supplementary Fig. S1), are uniformly sized, with minimal variability (498.1 ± 5.8 µm diameter) (Fig. 1a, and Supplementary Fig. S2). To closely recapitulate the 3D microenvironment of the human brain, we incorporated the neurospheroids with a Matrigel matrix. We counteracted the buoyancy of the neurospheroids in the microwells by casting first a thin layer of Matrigel. Following the mechanical removal of the excess above the microwells, a thin layer of Matrigel is formed on top of the array (Supplementary Fig. S1). Neuronal differentiation of the human neurospheroids in the ReN cell culture was initiated after 24–48 h by removing the growth factors including epidermal growth factor (EGF), and basic fibroblast growth factor (bFGF). For hiPSC culture, neuronal differentiation was induced by switching to a differentiation medium. The size of the neurospheroids in Matrigel increases during the initial self-aggregation stage of the ReN cells, in the first 48 h, and then remains constant over the course of one month culture (Fig. 1a). At the same time, the heterogeneity in size, which is large immediately after the start of ReN cell culture, decreases and the neurospheroids become mechanically confined inside the microwells (Fig. 1b). Consistent with previous reports that 3D cell cultures provide brain-like environment conditions^50,51^, we found that neuronal differentiation and neural network-like connections were presented in the neurospheroids array for both two-week-old ReN- and hiPSC-derived neurospheroids (Fig. 1c, and Supplementary Movies S1 and S2).

To recapitulate AD hallmarks *in vitro*, human ReN neural progenitor cells were developed according to our previous reports^38,39^ to express FAD mutations overexpressing human amyloid precursor protein (APP) and presenilin 1 (PSEN1). Control (ReN-G2) and FAD-derived (ReN-mGAP) neurospheroids were cultured in the array platform with Matrigel and differentiated over the course of 8 weeks (Fig. 1b–d, and Supplementary Movies S1 and S2). At 2-week after the start of differentiation in the array, we observed that processes extend to adjacent neurospheroids, both in ReN control (ReN-G2) and in ReN FAD (ReN-mGAP) as well as the hiPSC-derived neurospheroids (Fig. 1c), suggesting the formation of the neuronal network. After 8-week differentiation, the ReN-derived neurospheroids appear connected through robust and thick bundles of neurites documented by fluorescence imaging and scanning electron microscopy (Fig. 1c-d).

**Figure 2:**
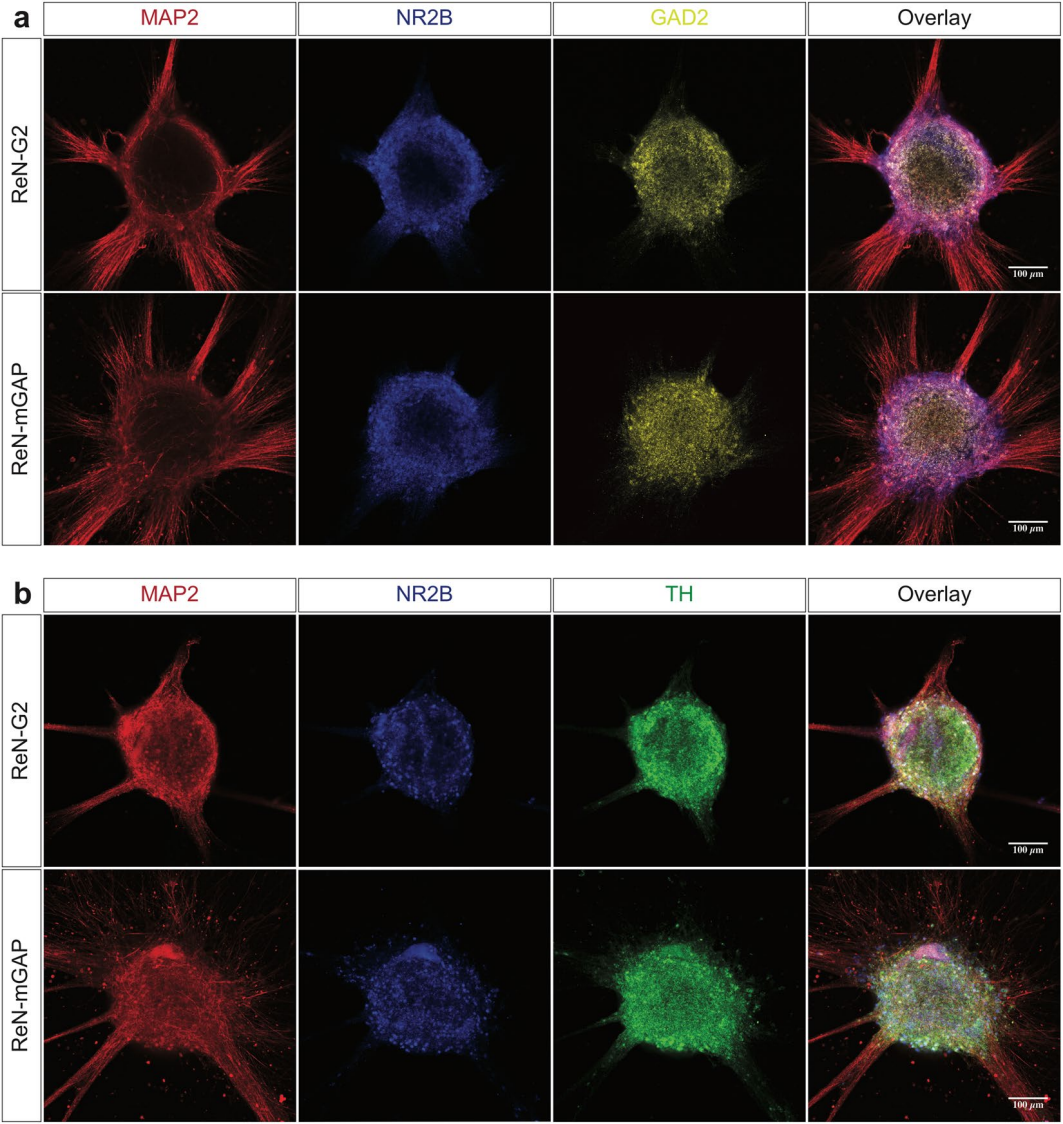
Expression of selected neuronal markers in ReN-derived neurospheroids. Two-month-old ReN-derived neurospheroids express MAP-2, microtubule-associated protein 2, NR2B, *N*-methyl D-aspartate receptor subtype 2B; GAD2, Glutamate decarboxylase 2 (**a**) and TH, tyrosine hydroxylase (**b**).

**Figure 3:**
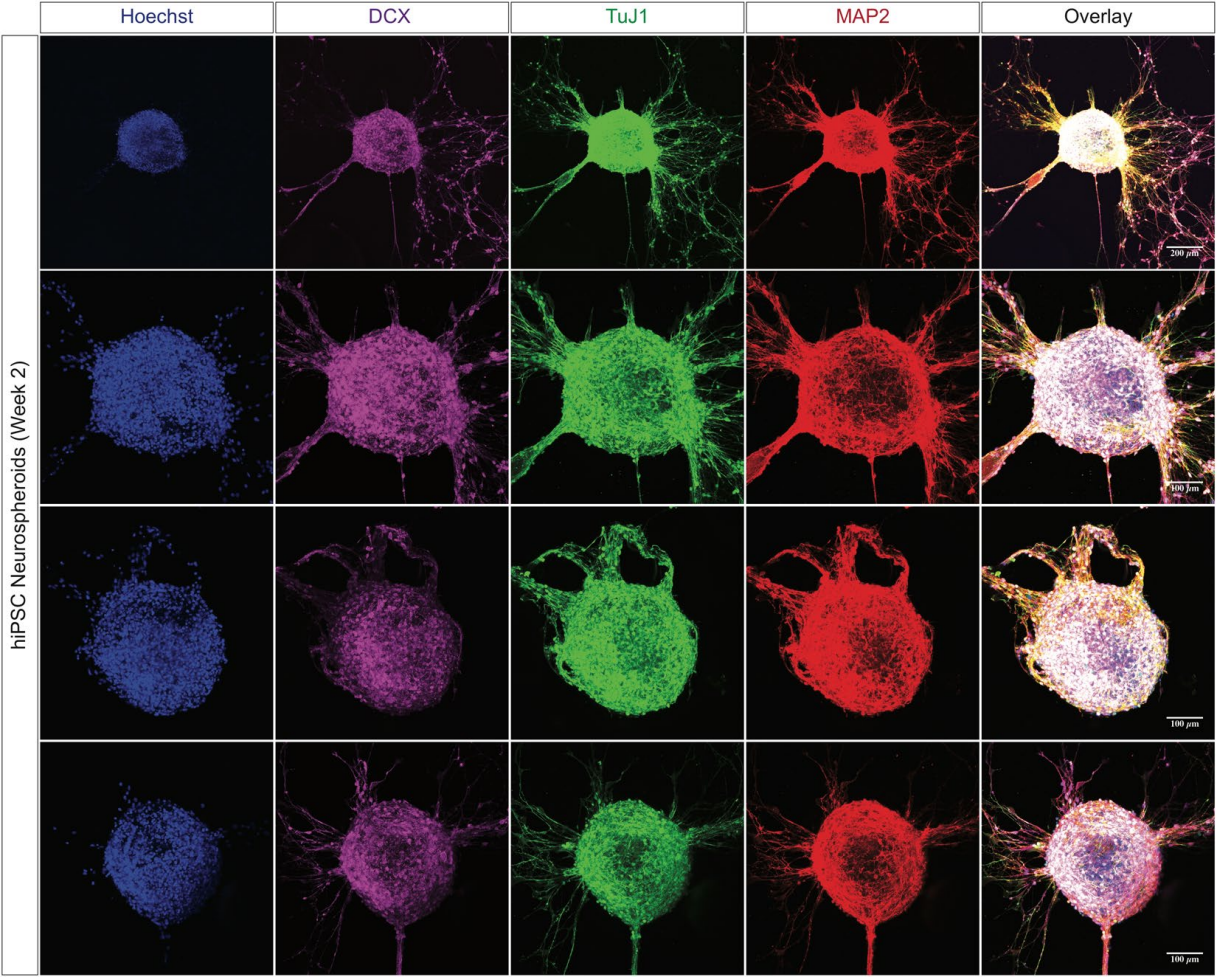
Generation and differentiation of hiPSC-derived neurospheroids in the 3D cell culture platform. Representative confocal images show expression of neuronal markers (MAP-2, microtubule-associated protein 2; TuJ1, ß-tubulin III; and DCX, doublecortin), and nuclei (Hoechst) in the hiPSC-derived neurospheroids at 2-week differentiation.

Two-month-old ReN-derived neurospheroids express different selected neuronal markers (MAP-2, microtubule-associated protein 2; NR2B, *N*-methyl D-aspartate receptor subtype 2B; GAD2, glutamate decarboxylase 2, and TH, tyrosine hydroxylase) (Fig. 2). To assess cell death rate/level and apoptotic cells number/level in the generated two-month-old ReN neurospheroids, we quantified the expression levels of cleaved caspase-3 (apoptosis marker) and Hoechst (nuclei marker). Z-stack series of confocal images showed a co-expression of positive cleaved caspase-3 and Hoechst in the center of the neurospheroids but the expression level of cleaved caspase-3 relatively low (Supplementary Fig. S3). The hiPSC-derived neurospheroids express multiple neuronal markers (TuJ1, ß-tubulin III; DCX, doublecortin, and MAP-2) at two weeks differentiation (Fig. 3). Analysis of the composite overlay of a series of Z-stack images from the hiPSC-derived neurospheroids to allow visualization of the entire neurospheroids displayed positive nuclei (Hoechst) in all focal points at two-week differentiation in the 3D culture platform.

**Figure 4:**
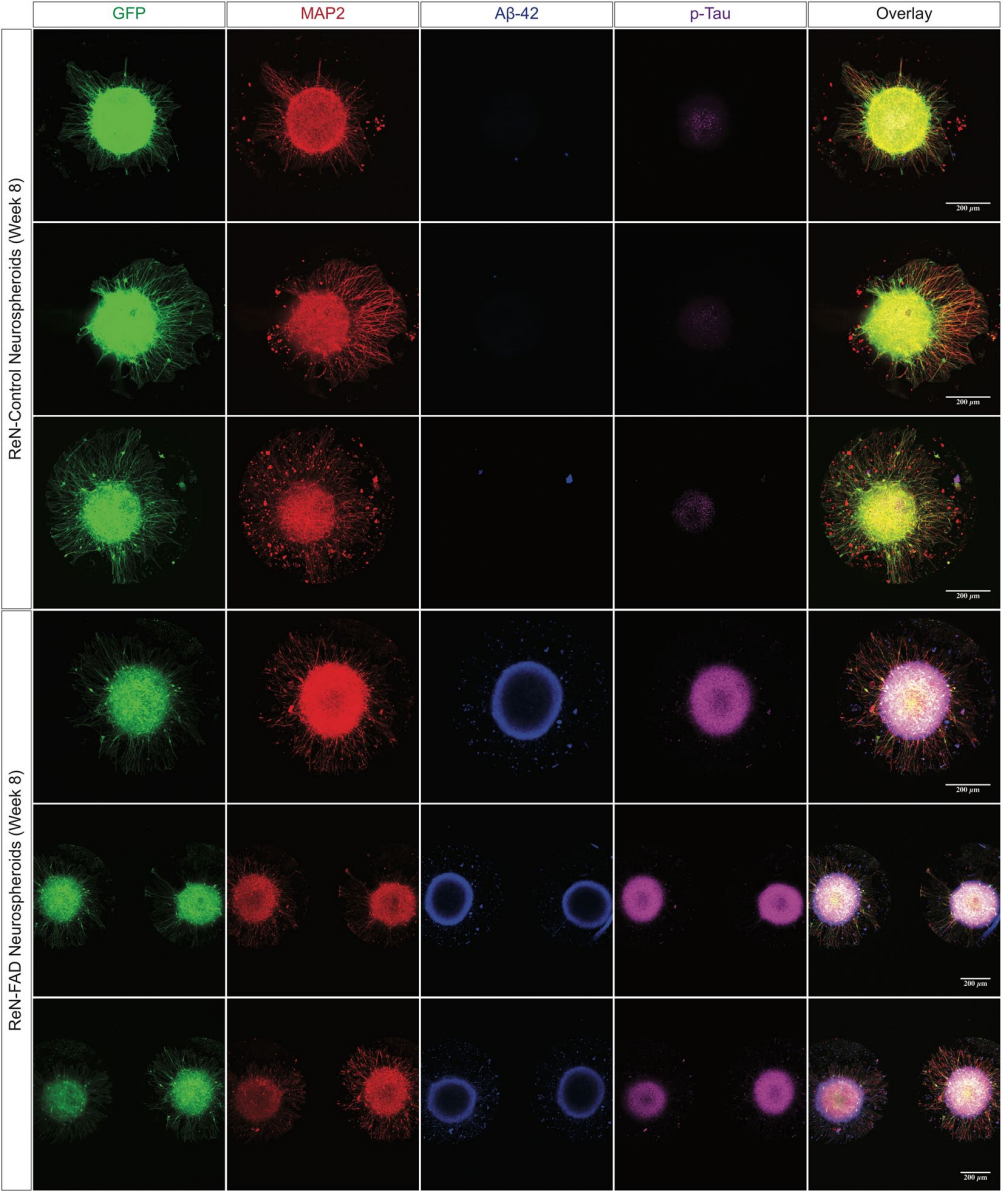
Reconstructing Alzheimer’s amyloid and tau pathology in 3D ReN-derived human neurospheroids. Representative confocal images show increased Aβ42 deposits and phosphorylated tau in FAD-derived neurospheroids after 8-week differentiation. Images show expression of the green fluorescent protein (GFP) in the engineered human neurospheroids, microtubule-associated protein 2 (MAP-2, neuronal marker), Aβ42 (isomer 1–42 Aβ), and p-Tau (phosphorylated tau marker).

**Figure 5:**
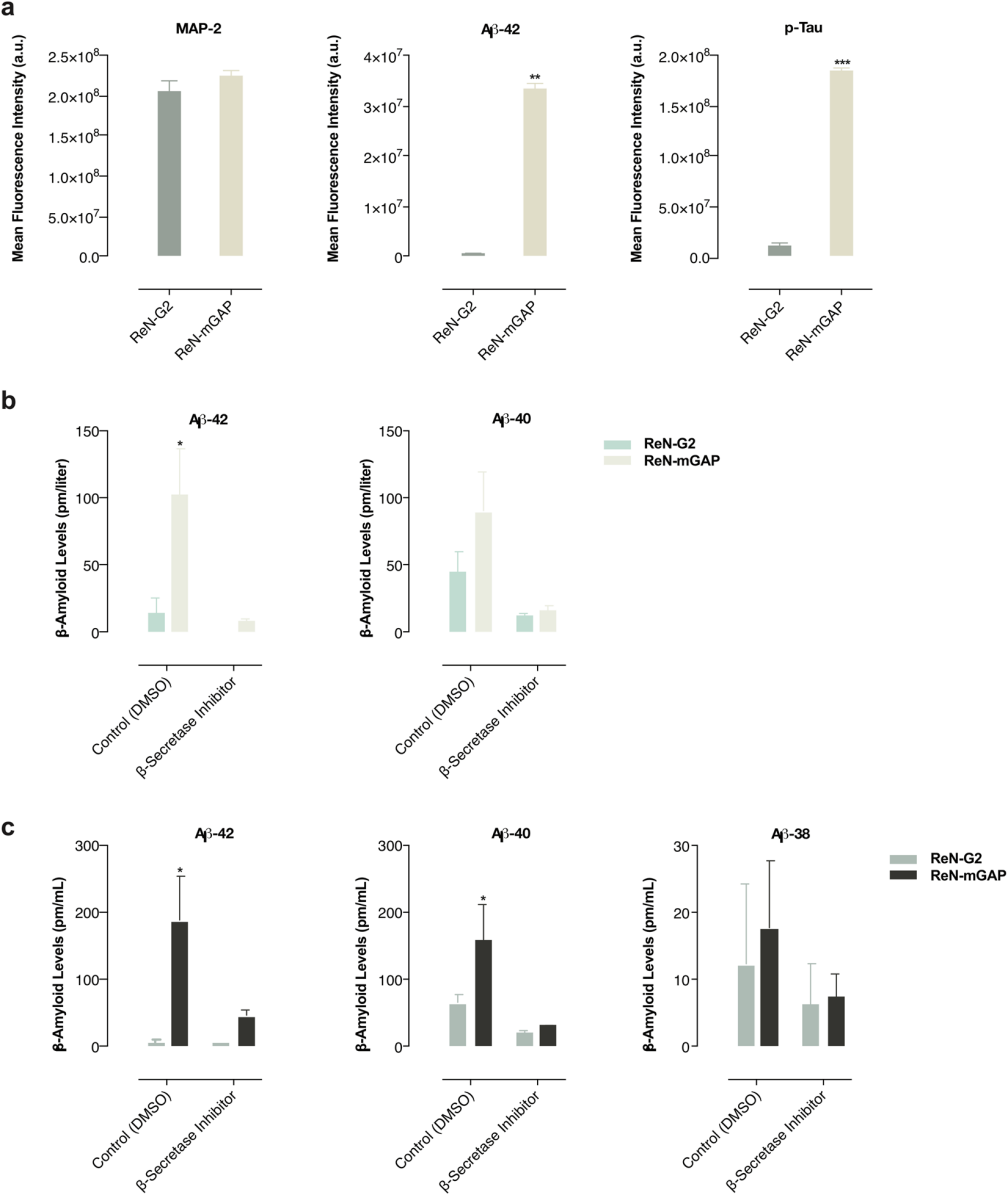
Quantification of changes in amyloid-β and phosphorylated tau in 3D ReN-derived neurospheroids. (**a**) Graphs show a quantitative comparison of immunohistochemical staining measured by mean fluorescence intensity of MAP-2, Aβ42, and p-tau levels. (**b**,**c**) Graphs show higher Aβ levels in FAD-derived neurospheroids (ReN-mGAP) compared to control ReN-G2 as measured by Aβ ELISA kit (**b**) and MesoScale Discovery 96-well Mouse Pro-Inflammatory V-PLEX Assay (**c**). Treatment with 1 µM β–secretase inhibitor (LY2886721) decreased Aβ38, Aβ40 and Aβ42 levels in both ReN-G2 control and ReN-mGAP FAD cell line. *P < 0.05; **P < 0.01; ***P < 0.001; ****P < 0.0001; ANOVA followed by a post hoc Tukey’s test; means ± SEM; n = 4 per each sample.

To detect and analyze AD markers in FAD-derived human neurospheroids, the neurospheroids were fixed with 4% PFA after 8-week differentiation and stained for Aβ and p-tau, the Aß levels in the cell media were measured by Aβ ELISA kit and MesoScale Discovery V-PLEX Assay. As previously reported, FAD-derived neurospheroids (ReN-mGAP) showed an increase in Aβ isoforms including Aβ42, Aβ40, Aβ38, and accumulation of total and phosphorylated tau (Figs 4 and 5a and Supplementary Figs S4 and S5). When we treated the FAD neurospheroids (ReN-mGAP) with a β-secretase inhibitor (LY2886721), all Aβ isoforms (Aβ38, Aβ40, Aβ42) in the supernatant cell culture media above the array were significantly decreased compared to control, dimethyl sulfoxide (DMSO) treated neurospheroids (Fig. 5b,c and Supplementary Fig. S6).

**Figure 6:**
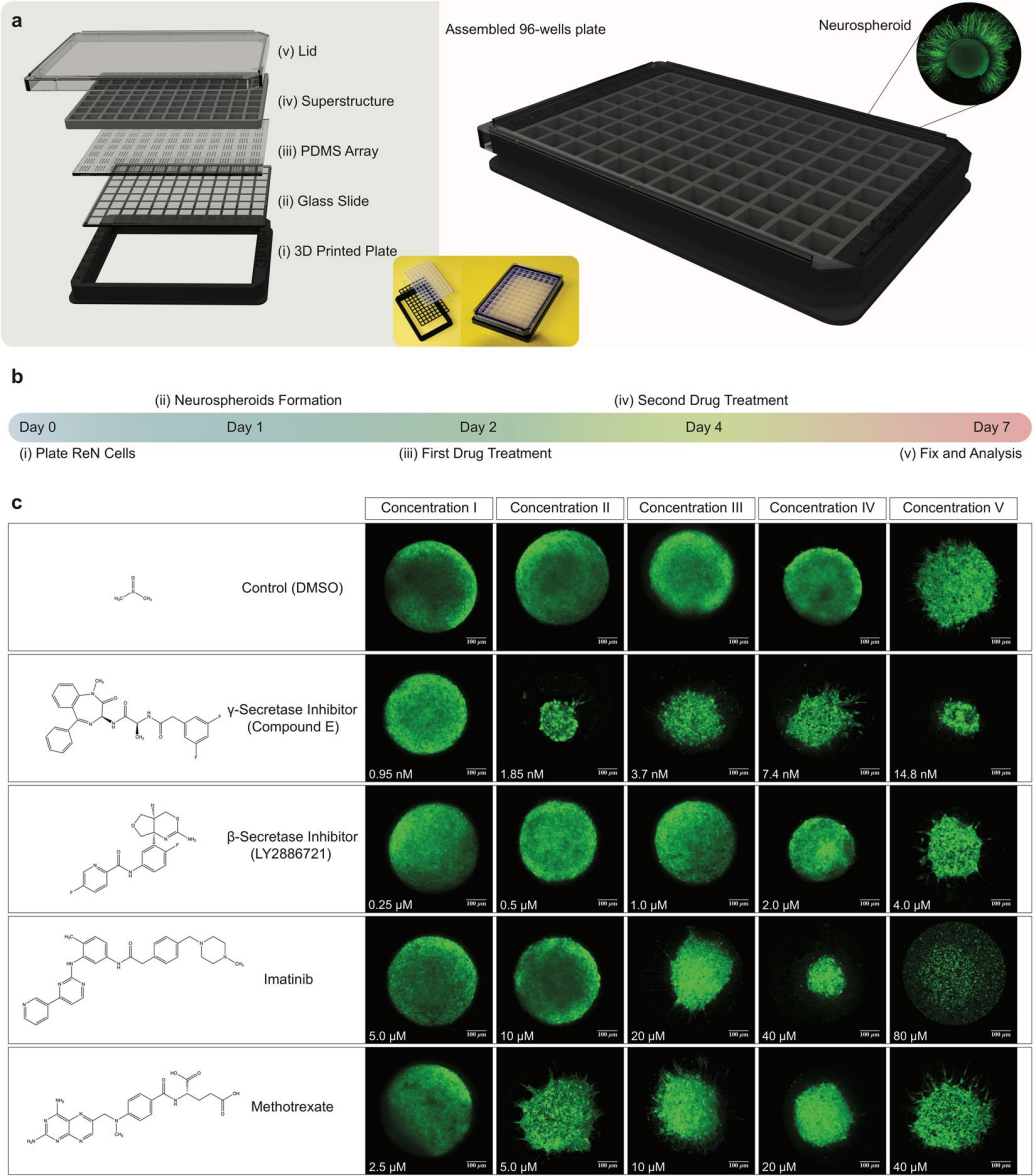
Platform assembly and qualitative evaluation of the ReN-derived neurospheroids in the 3D platform under the effect of active compounds. (**a**) The microfabricated 96-well cell culture plate with 1,536 microwells for neurospheroids culture stacks the PDMS array between a glass slide and a plastic superstructure. The assembly is mounted inside a 3D printed plate and covered with a lid. (**b**) The diagram shows the timeline of the neurospheroid growth, drug treatment, and analysis protocols. (**c**) Confocal representative images show a qualitative comparison of the neurospheroids size and morphology at day seven after treatment with DMSO (control), γ–secretase inhibitor (Compound E), β–secretase inhibitor (LY2886721), Methotrexate, and Imatinib at five different concentrations.

To further explore the capability of our neurospheroids array platform to accelerate drug screening, we leveraged microfabrication and 3D printing techniques to develop a 96-well cell culture plate with 1,536 microwells. The 96-well plate is comprised of five main components: (i) a 3D designed and printed 96-well frame, (ii) a high-quality commercial glass substrate with high transmittance of over 92% and high optical clarity for fluorescence wavelengths, (iii) a microfabricated array with 1,536 microwells (500 µm diameter and 600 µm depth), (iv) a commercial self-adhesive 96-well silicone superstructure that adheres to the array, and (v) a lid (Fig. 6a). Using the 96-well array plate, we generated neurospheroids and treated these with various compounds including γ–secretase inhibitor (Compound E), β-secretase inhibitor (LY2886721), Imatinib, and Methotrexate. Neurospheroids were monitored at five different concentrations for each compound over the course of one week (Fig. 6b). DMSO treated neurospheroids were employed as a control. We noted a 20–30% decrease in neurospheroid diameter for the two highest DMSO concentrations. Neurospheroids treated with γ–secretase inhibitor, Methotrexate, and Imatinib displayed neurite-like projections (Fig. 6c, and Supplementary Fig. S7). Previously, we showed that γ–secretase inhibitor treatments accelerated the neuronal differentiation and neurite-like projections possibly by blocking the Notch signaling, in human neurospheroid cultures^45^. As expected, the neurospheroids treated with ${\rm{\beta }}$–secretase inhibitor did not show any neurite-like projections (Fig. 4c, and Supplementary Fig. S7). The neurospheroids treated with γ–secretase inhibitor, Methotrexate, and Imatinib also showed a smaller size than the DMSO treated controls (Fig. 4c, and Supplementary Fig. S7).

**Figure 7:**
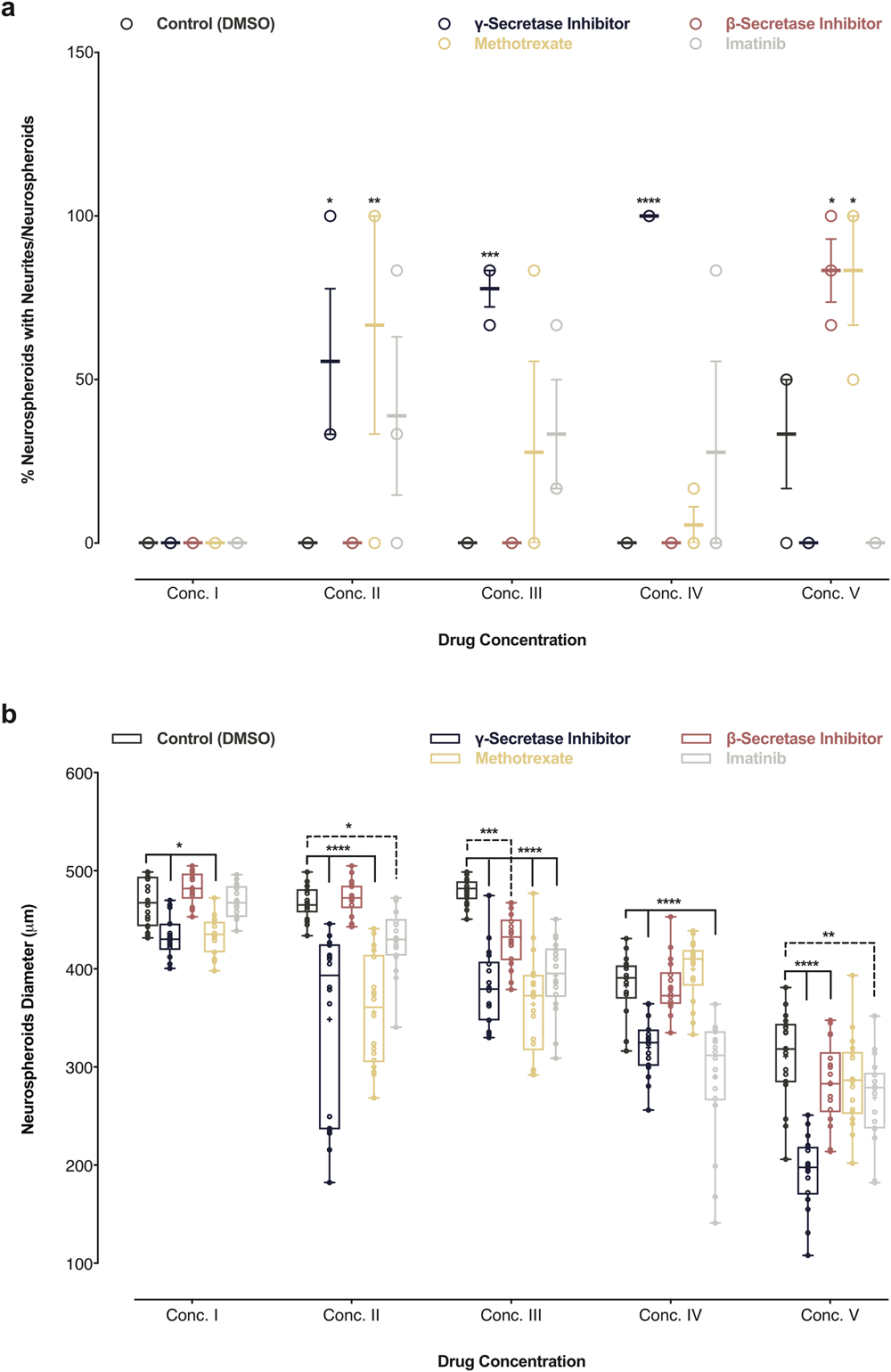
Quantification of the effect of γ–secretase inhibitor, β–secretase inhibitor, Methotrexate, and Imatinib treatments on homogenously-sized neurospheroids generated in a 3D array platform. (**a**) The effect of γ–secretase inhibitor (Compound E), β–secretase inhibitor (LY2886721), Methotrexate, Imatinib, and DMSO (control), on neurospheroids neurites at day seven. (**b**) The effect of γ–secretase inhibitor (Compound E), β–secretase inhibitor (LY2886721), Methotrexate, Imatinib, and DMSO (control), on the neurospheroids diameter at day 7. *P < 0.05; **P < 0.01; ***P < 0.001; ****P < 0.0001; ANOVA followed by a post hoc Dunnett’s test; means ± SEM; n = 6 per each sample.

In addition to the qualitative changes noted by monitoring the shape and homogeneity of the neurospheroids, we also quantified the formation of neurites (Fig. 7a, and Supplementary Fig. S8) and the changes in neurospheroid diameter (Fig. 7b, and Supplementary Fig. S8). After five days of exposure to the compounds, we noted a persistent increase in the fraction of neurospheroids with neurite-like projections and a decrease in neurospheroid diameter with increasing concentrations of γ–secretase inhibitor (Compound E). These changes were minimal in the presence of the β–secretase inhibitor (LY2886721), except for the highest concentrations. The fraction of neurospheroids with neurites after exposure to Methotrexate and Imatinib was highest at concentration II and then decreased at higher concentrations. While the diameter of the neurospheroids was reduced, the effect was consistent with the toxicity of Methotrexate and Imatinib at concentrations above II.

## Discussion

We designed a platform that holds neurospheroids inside microwells-based arrays and employed this tool to replicate key features of the Alzheimer’s disease phenotype *in vitro*. We leveraged microscale technologies to fabricate microwells and generate homogenously-sized neurospheroid arrays derived from immortalized human neural progenitor cells (NPCs) as well as hiPSC-derived neural stem cells (NSCs). We added Matrigel to the microwells to hold the newly formed neurospheroids and initiate the differentiation of the neural stem cells into neurons, astrocytes, and other types of non-neuronal cells. Adding Matrigel to the microwells is challenging due to the intrinsic low adherence characteristic of Matrigel hydrogel to polydimethylsiloxane (PDMS) surface chemistry and leads to disorganization of the neurospheroids from their microwell during medium exchanges. We overcame these challenges by removing the excess of Matrigel hydrogel after solidifying. The resulting uniform thin layer of extracellular matrix covers the neurospheroids in the array as well as the array surface and allows the neurospheroids to differentiate for several weeks.

During differentiation, hiPSC-derived neurospheroids express neuronal markers including ß-tubulin III (TuJ1), doublecortin (DCX), and mature microtubule-associated protein 2 (MAP-2). We also show the multiple adult neuronal type markers in the 3D cell culture platform including NR2B (excitatory), GAD2 (inhibitory), and tyrosine hydroxylase (dopaminergic neurons) in 3D differentiated neurospheroids from ReN cells. This is in line with our previous report that we found ReNcell VM cells differentiated in 3D culture conditions expressed excitatory, inhibitory and dopaminergic markers, confirming the heterogeneous nature of neurons derived from ReNcell VM^38^. Since mostly hippocampal and cortical neurons are affected in AD, the presence of TH-positive dopaminergic neurons may limit the applications of our 3D ReN cell model in studying forebrain-specific mechanisms.

One of the interesting aspects of this new model is that the neurospheroids display thick bundles of dendrites extending outward, different from the current brain organoid models, which show dendrites extending inward. This unique design can also accommodate extensive neurite outgrowth and neural networks formation between adjacent neurospheroids. While the neurospheroids are confined to microwells, they can be imaged repeatedly and indexed based on their location on the plate.

For *in vitro* study of AD pathogenesis and drug treatments, our neurospheroids array tool: (i) provides a platform that helps monitor individual, uniformly-sized neurospheroids during long-term culture, (ii) incorporates extracellular matrix proteins that mimic the human brain 3D microenvironment, (iii) takes advantage of genetically-engineered human neural stem cells that produce high level of pathogenic Aβ as well as physiologically-relevant hiPSC, and (iv) is compatible with high-throughput machines for drug screening. Using this platform, we showed that the FAD neurospheroids derived from immortalized ReN cells recapitulate major hallmarks of the AD including the accumulation of extracellular Aβ aggregates and intracellular p-tau. Moreover, we also observed that the levels of both total and phosphorylated tau were increased in the FAD-derived neurospheroids compared with controls (*data not shown*). This observation is consistent with recent studies using FAD patient-derived neurons, which showed the increase in both total tau and phosphorylated tau at the early stage of tauopathy (termed “tau proteostasis”)^22^. We also demonstrated the capability of the system to block the release of pathogenic and non-pathogenic Aβ isoforms by ${\rm{\beta }}$–secretase inhibitor. Other genetically-engineered cells may potentially be used for the study of AD and other neurodegenerative diseases.

Although our FAD neurospheroids model nicely recapitulated the accumulation of amyloid-β and phospho tau, our 3D culture models, as well as a majority of the current cellular and animal AD models, require overexpression of FAD genes with single or multiple mutations. Since most of AD patients (>95%) do not carry either FAD mutations nor FAD gene overexpression (sporadic AD), current cellular AD models with FAD mutations may not be suitable for modeling sporadic AD patients. Even iPSC-derived neurons from FAD patients cannot be free from this criticism, since they harbor genetic mutations that represent the very small percent of AD patients. We believe that developing 3D AD neurospheroid models without carring rare FAD mutations, will be an important next step in comprehensively recapitulating AD pathogenic cascade in cellular models.

To validate the use of the neurospheroid arrays as drug screening tools compatible with high-throughput screening machines, we developed a new 96-well platform. Using this platform, we tested the quantitative and qualitative changes in neurospheroids morphology, size and neurite extension in the presence of multiple compounds including the inhibitors of ${\rm{\beta }}$– and γ–secretases, which have been primary targets for AD drug discovery. We found that γ–secretase inhibitor (Compound E) induced abnormal neuronal differentiation in our 3D neurospheroids derived from ReN cells, in the presence of growth factors in the culture medium (EGF, bFGF), which they block the neuronal differentiation of the neurospheroids, similar to our previous study^45^. These results, suggest that the therapeutic inhibition of γ–secretase activity may interfere with adult neural stem cell function in patients’ brains. In contrast, ${\rm{\beta }}$–secretase inhibitor (LY2886721) did not show any evidence of toxicity. One interesting finding of the current drug screening study is the similar trend of cell toxicity showed by all tested compounds at the highest concentration. Part of this effect is likely due to the toxicity of DMSO on neurospheroids, consistent with other reports of DMSO toxicity on neuronal cells^52^. Moreover, we found that Methotrexate^53,54^ and Imatinib^55,56^ at higher concentrations exert a potent effect on neurite-like projections and potential neural differentiation in the human neurospheroids. This is in line with previous findings showing that Methotrexate is neurotoxic to neural progenitor cells and human embryonic stem (ES) cells by inhibiting proliferation while inducing neuronal differentiation on ES and progenitor cells in the hippocampus^57,58^. To the best of our knowledge, the effect of Imatinib on human neural progenitor cells and in particular 3D neuronal brain models such as neurospheroids has not yet been reported. More systematic studies are needed to get a deeper understanding of the mechanism of these compounds on neural progenitor cells and hiPSC. Overall, this unique platform may serve as a reliable, physiologically-relevant human brain cell culture model, and cost-effective high-throughput drug screening for not only AD but also for other neurodegenerative diseases.

## Methods

### Cell culture

ReNcell VM human neural progenitor (Cat. No. SCC008), and human iPSC-derived neural progenitor cells (Cat. No. SCC035) were purchased from EMD Millipore, USA. A familial Alzheimer’s disease (FAD) cell line that exhibits substantial amyloid pathology was engineered according to the previous reports^38,39^. In brief, immortalized human neural progenitor cell line ReNcell VM (ReN) were transfected with internal ribosome entry site (IRES)-mediated polycistronic lentiviral vectors containing FAD-related genes encoding human amyloid precursor protein (APP) with both the K670N/M671L (Swedish) and V717I (London) mutations (APPSL), presenilin 1 (PSEN1) with the ΔE9 mutation (PSEN1(ΔE9)) with both GFP and mCherry as reporters for viral infection. Fluorescence-activated cell sorting (FACS) was then used to enrich the population of cells with the highest expression levels. The ReN and hiPSC cells were cultured in Matrigel-coated T25 culture flasks (1:100 dilution of Matrigel:DMEM-F12 medium) using ReN cell proliferation medium, or ENStem-A^TM^ neural expansion medium. ReN cells proliferation medium was prepared by combining 484.5 mL DMEM/F12 medium (Gibco/Life Technologies) with 0.5 mL heparin (STEMCELL Technologies), 10 mL B27 (Gibco/Life Technologies), 5.0 mL of 100X penicillin/streptomycin/amphotericin B (Lonza), 0.5 mL epidermal growth factor (EGF, 20 μg/mL, Sigma-Aldrich), and 0.4 mL basic fibroblast growth factor (bFGF, 25 μg/mL, Stemgent). Human iPSC expansion medium was prepared by combining 99 mL ENStem-A neural expansion medium (Cat. No. SCM004, EMD Millipore) with 1.0 mL EmbryoMax L-Glutamine solution (100X), 200 mM (Cat. No. TMS-002-C, EMD Millipore) and 1.0 mL EmbyroMax ES Cell-Qualified Penicillin-Streptomycin solution, 100X (Cat. No. TMS-AB2-C, EMD Millipore). FGF-2 (Cat. No. GF003, EMD Millipore) was added to the ENStem-A neural expansion medium fresh to the final concentration of 40 ng/mL during media exchanges.

### Fabrication of the array platform

The masters for preparing the PDMS arrays (500 μm diameter and 600 μm depth) were designed and microfabricated by patterning SU-8 photoresist (MicroChem Co.) on a silicon wafer using standard photolithography techniques^59^. Devices presenting 5 × 5 microwell arrays were bonded after plasma treatment to uncoated 6-well glass bottom plate (MatTek Corporation). The 96-well platform for drug screening is composed of five components: (i) a 3D designed and printed 96-well frame, (ii) a high-quality glass (Nexterion, Applied Microarrays, Inc.) with high transmittance of over 92% and high optical clarity for fluorescence wavelengths, (iii) a microfabricated PDMS microwell array, (iv) a 96-well silicon superstructure (Nexterion, Applied Microarrays), and (v) a lid. The microplate frame was designed and printed using a MakerBot Replicator 2 desktop 3D printer. The master for preparing the PDMS array with 1,536 microwells (500 μm diameter and 600 μm depth) was designed and microfabricated with SU-8 photoresist (MicroChem Co.) on a silicon wafer. The PDMS array was molded by casting the liquid prepolymer composed of a mixture of 10:1 silicon elastomer and a curing agent (Sylgard 184). The mixture was cured at 75 °C for 12 h, and then the PDMS mold was peeled from the silicon wafer. The molded PDMS block was turned upside down and bonded to the glass substrate using oxygen-plasma treatment. Finally, the self-adhesive 96-well silicon superstructure adhered to the 96-well PDMS array and all components were assembled into one 96-well plate.

### Generation of human 3D neurospheroids from stem cells

Neurospheroids were formed by plating 30 × 10^6^ cells/mL of the neural progenitor cells derived from ReNcell VM cell (ReN-G2, control cell line; and ReN-mGAP, FAD cell line) or hiPSC in expansion medium on top of the array. After 20 min, expansion medium was added on the side of the array, and then half of the medium was aspirated to remove floating cells from the device. The devices were incubated at 37 °C and 5% CO_2_ atmosphere. After 24 h, the proliferation medium was changed. After 48 h required for complete neurospheroids formation in each well, the proliferation media was aspirated, and Matrigel at a dilution of 1:4 in differentiation medium (ReN-derived neurospheroids) and in 1:1 expansion:differentiation medium (hiPSC-derived neurospheroids) was applied on the top of the array to cover the whole surface area. After 24 h required for solidifying Matrigel at 37 °C, the Matrigel on the surface of the devices was removed gently using a gel loading tip-tilted into 90 degrees, and the medium was replaced with fresh differentiation medium. The cell culture was maintained up to eight weeks, changing the differentiation medium every 4 days. ReN cells differentiation medium was prepared by combining 484.5 mL DMEM/F12 medium with 0.5 mL of heparin, 10 mL of B27 and 5.0 mL of 100X penicillin/streptomycin/amphotericin B. ENStem-A neural differentiation medium (Cat. No. SCM017, EMD Millipore) was used for differentiation of human iPSC-neural progenitor cells.

### Immunofluorescence staining

Neurospheroids were cultured up to eight weeks in differentiation medium followed by rinsing in phosphate-buffered saline and fixation in 4% paraformaldehyde (Electron Microscopy Sciences) for overnight at room temperature and then washed three times with 1X Tris Buffered Saline with Tween 20 (TBS-T). The neurospheroids were immersed for overnight at 4 °C in blocking solution. Blocking/dilution solution for immunostaining was prepared by adding 2.5 g of BSA (Sigma-Aldrich), 5.63 g of glycine and 0.25 g of gelatin in 200 mL of TBS-T and heated at 55 °C for ~10 min to allow gelatin to dissolve. 10 mL of donkey serum (Sigma-Aldrich) and TBS-T was added to make the final volume of 250 mL. The blocking/dilution solution was then filtered with a 0.4 μm filter unit (Gibco) and stored at 4 °C. The blocking step was followed by permeabilization with 0.1% Triton X-100 for 45–60 min at room temperature. The cells were washed three times with 1X TBS-T and the neurospheroids were incubated with primary antibodies in blocking solution at 4 °C overnight followed by species-specific secondary antibodies at 4 °C overnight in the dark (Table S1). The cell nuclei were stained with Hoechst 3342 (1:2000 dilution) for 20 min at room temperature. Following staining of the neurospheroids, a series of images were captured using the Z-stacking function on an inverted Nikon Eclipse Ti microscope (Nikon Instruments), to allow visualization of the entire neurospheroids. The excitation wavelengths of mCherry and anti-chicken Alexa fluor 568 antibodies, used for MAP2 staining used for in this study, partially overlap. However, the major differences in signal strength allowed us to differentiate the MAP2 signal versus Alexa fluor 568. All images were processed using Fiji (ImageJ) software.

### Electron microscopy

For Scanning Electron Microscopy (SEM), ReN-derived neurospheroids were fixed in 2.5% glutaraldehyde for 30 min, washed three times with PBS, and then post-fixed in aqueous 1% OsO_4_ for 1 h. After washing in PBS three times for 15 min each, they were dehydrated through a graded series of ethanol in PBS (30 to 90%) for 15 min each and washed with absolute ethanol three times before they were dried in hexamethyldisilazane solution and allowed to air dry under safety hood. Afterwards, the arrays with fixed neurospheroids were mounted onto aluminum stubs, sputter-coated with 5 nm of platinum/palladium, and imaged in an Ultra55 Field Emission Scanning Electron Microscope (FESEM) at 10 kV with In lens SE detector.

### Drug treatments

Neurospheroids were formed by seeding neural progenitor ReN-G2 (12 × 10^6^ cells/mL in proliferation medium) on top of the array. After 20 min, proliferation medium was added on the side of the array, and then half of the medium was aspirated from the side to remove floating cells from the device. The devices were incubated at 37 °C and 5% CO_2_ atmosphere. After 48 h, the fully formed neurospheroids in the 96-well plate were treated with different drugs in five different concentrations. The ReN-derived neurospheroids were maintained for one week, changing the medium with fresh differentiation medium including the drugs after four days. After one week, neurospheroids were rinsed with phosphate-buffered saline and fixed with 4% paraformaldehyde for overnight at 4 °C and then washed three times with D-PBS. The fixed neurospheroids were imaged using inverted Nikon Eclipse Ti microscope (Nikon Instruments).

### Quantitative measurement of changes in Amyloid-β

The measurement of Aβ38, 40, and 42 levels was performed using two different assays: (i) amyloid-β ELISA—Aβ40 and Aβ42 levels were measured by the Human Amyloid-β ELISA Kit (Wako Pure Chemicals, Osaka, Japan). The conditioned media from differentiated ReN neurospheroids were collected and diluted by 1:2 with a dilution buffer provided by the company. The Synergy 2 ELISA plate reader (BioTek Instruments, Winooski, VT, USA) was used to quantify Aβ40 and Aβ42 ELISA signals. (ii) Aβ38, 40 and 42 levels were measured by MesoScale Discovery (MSD, Rockville, MD) 96-well Mouse Pro-Inflammatory V-PLEX Assay as outlined in the manufacturer’s protocol. Briefly, 150 μl of Diluent was added to the plate coated with an array of Aβ capture antibodies and incubated at room temperature with shaking for one hour, followed by washing with wash buffer (provided in the kit). A volume of 25 μL of the detection antibody solution plus 25 μL of prepared samples were added and incubated for 2 h with vigorous shaking at room temperature. The plate was washed with wash buffer before adding 150 μL of 2X Read Buffer T, and immediately read on a Meso QuickPlex SQ 120.

### Statistical analysis

All statistical analyses were performed using GraphPad Prism 7. All results are presented as box plots, with means, standard error of the mean, 5 and 95% percentiles and outliers. Quantitative analysis of cell staining for a particular marker in the neurospheroids were performed using Fiji (ImageJ) software. The effects of various drugs on neurospheroids size and neurites at different concentrations were analyzed using ANOVA with Dunnett’s multiple comparisons test. Quantitative analysis of Aβ expression was analyzed using ANOVA with Tukey’s multiple comparisons test. Differences between means were considered significant at *P < 0.05; **P < 0.01; ***P < 0.001; ****P < 0.0001.

### Data availability

All data generated or analyzed during this study are included in this published article (and its Supplementary Information files).

## Electronic supplementary material

Supplementary information

Supplementary Movie S1

Supplementary Movie S2
