# Supplementary material for: Human Neurospheroid Arrays for In Vitro Studies of Alzheimer’s Disease
Source: Sci Rep. 2018 Feb 5;8:2450. doi: 10.1038/s41598-018-20436-8 (PMC5799361; doi:10.1038/s41598-018-20436-8)
Supplement: Supplementary file 1 — Supplementary information [file 41598_2018_20436_MOESM1_ESM.pdf]

SUPPLEMENTARY INFORMATION

Human Neurospheroid Arrays for *In Vitro* Studies of Alzheimer's Disease

Mehdi Jorfi,[1,3] Carla D'Avanzo,[2,3] Rudolph E. Tanzi,[2] Doo Yeon Kim,[2*] and Daniel Irimia[1*]

[1]Center for Engineering in Medicine, Department of Surgery, Massachusetts General Hospital, Harvard Medical School, Charlestown, Massachusetts 02129, United States

[2]Genetics and Aging Research Unit, MassGeneral Institute for Neurodegenerative Disease, Massachusetts General Hospital, Harvard Medical School, Charlestown, Massachusetts 02129, United States

[3]These authors contributed equally to this work.

Correspondence and requests for materials should be addressed to:

Doo Yeon Kim, Ph.D.
Assistant Professor of Neurology
Genetics and Aging Research Unit
Massachusetts General Hospital
Harvard Medical School
114, 16th street, Charlestown, MA 02129
Tel. 617-724-1505, Fax. 617-724-1823
Email: dkim@helix.mgh.harvard.edu

and

Daniel Irimia, M.D., Ph.D.
Associate Professor
Department of Surgery
Massachusetts General Hospital
Harvard Medical School
Associate Director, BioMEMS Resource Center
114, 16th street, Charlestown, MA 02129
Tel: 617-724-6543, Fax: 617-724-2999
Email: dirimia@mgh.harvard.edu

Table S1. Primary and secondary antibodies used for immunofluorescence staining.

**Marker**	**Antibody**	**Host**	**Supplier**	**Cat. No.**	**Dilution**
Neuron (dendritic)	Anti-MAP2	Chicken	EMD Millipore	AB5543	1:500
Neuron (neuritic)	Anti-Tuj1	Rabbit	Sigma	T2200	1:100
Neuron (neuritic)	Anti-DCX	Mouse	Abcam	Ab135349	1:200
Neuron (synaptic)	Anti-NR2B	Mouse	NeuroMab	N/59/36	1:2
Neuronal	Anti-GAD2	Rabbit	Cell Signaling Technology	D5G2	1:50
Neuron (dopaminergic)	Anti-tyrosine hydroxylase	Rabbit	Cell Signaling Technology	2792S	1:50
p-tau	AT-8	Mouse	ThermoFisher Scientific	MN1020	1:30
Total tau	Anti-tau	Rabbit	DAKO	A0024	1:500
Total tau	Tau46	Mouse	Cell Signaling Technology	4019	1:100
Aβ	3D6	Mouse	A gift from Lilly	NA	1:400
Aβ 40	β-amyloid (1-40 specific)	Rabbit	Cell Signaling Technology	D8Q71	1:400
Aβ 42	β-amyloid (1-42 specific)	Rabbit	Cell Signaling Technology	D9A3A	1:200
Apoptosis	Cleaved Caspase-3	Rabbit	Cell Signaling Technology	D175	1:200
CF^{TM}405M Secondary Ab	Anti-mouse	Goat	Biotium	A-31556	1:200
Alexa Fluor 488 Secondary Ab	Anti-rabbit	Goat	Abcam	Ab150077	1:200
Alexa Fluor 568 Secondary Ab	Anti-chicken	Goat	Life Technology	A-11041	1:400
Cy5 Secondary Ab	Anti-mouse	Donkey	Jackson ImmunoResearch	715-175-150	1:200
Cy5 Secondary Ab	Anti-rabbit	Donkey	Jackson ImmunoResearch	711-175-152	1:200

MAP-2, microtubule-associated protein 2; Tuj1, beta-tubulin III; DCX, Doublecortin; NR2B, *N*-methyl D-aspartate receptor subtype 2B; GAD2, Glutamate decarboxylase 2; NA, not applicable.

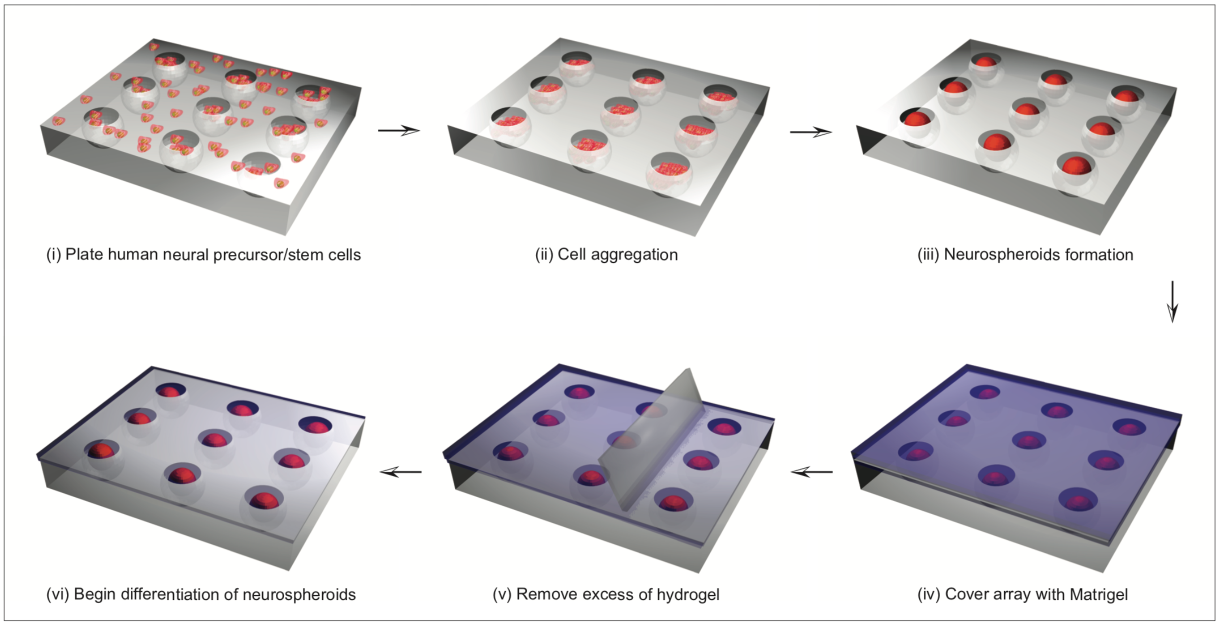

Figure S1. Generation of human neurospheroids in 3D platform. Schematics show the human neurospheroids generation and differentiation process in the 3D microfabricated platform.

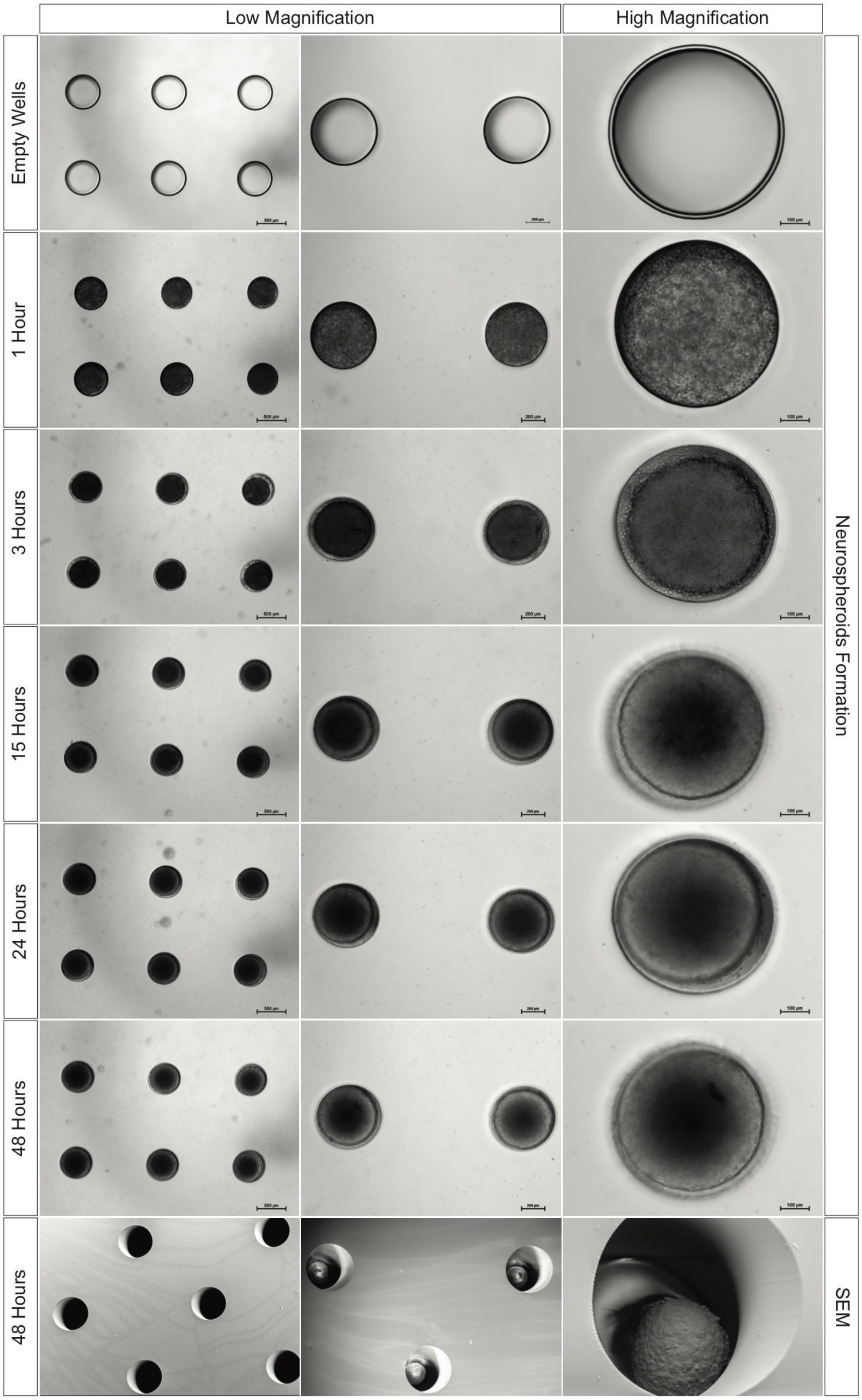

Figure S2. Formation of human neurospheroids in 3D microwell array. Phase contrast and scanning electron microscopy (SEM) images show the formation of neurospheroids over the course of 24 hours after seeding ReN cells.

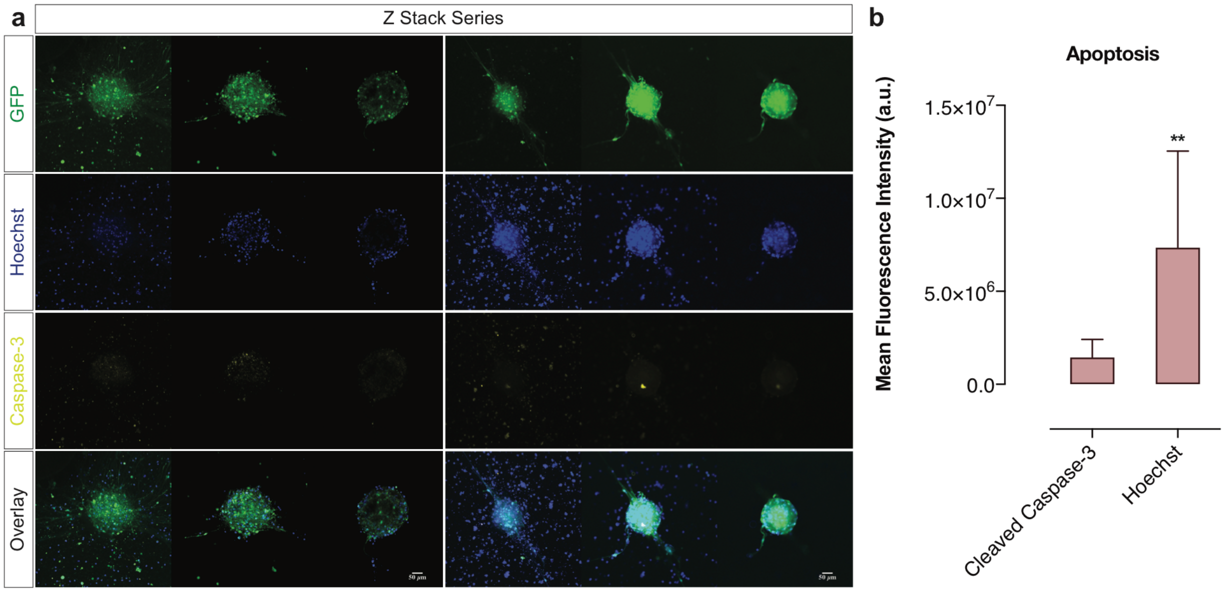

Figure S3. Quantification of apoptotic cells in the human neurospheroids. (a) Representative confocal z-stack image series show the co-expression of the apoptosis marker, cleaved caspase-3 and nuclei marker (Hoechst) in two-month-old neurospheroids generated in the 3D array platform. (b) The graph shows a quantitative comparison of immunohistochemical staining measured by mean fluorescence intensity of cleaved caspase-3 and Hoechst.

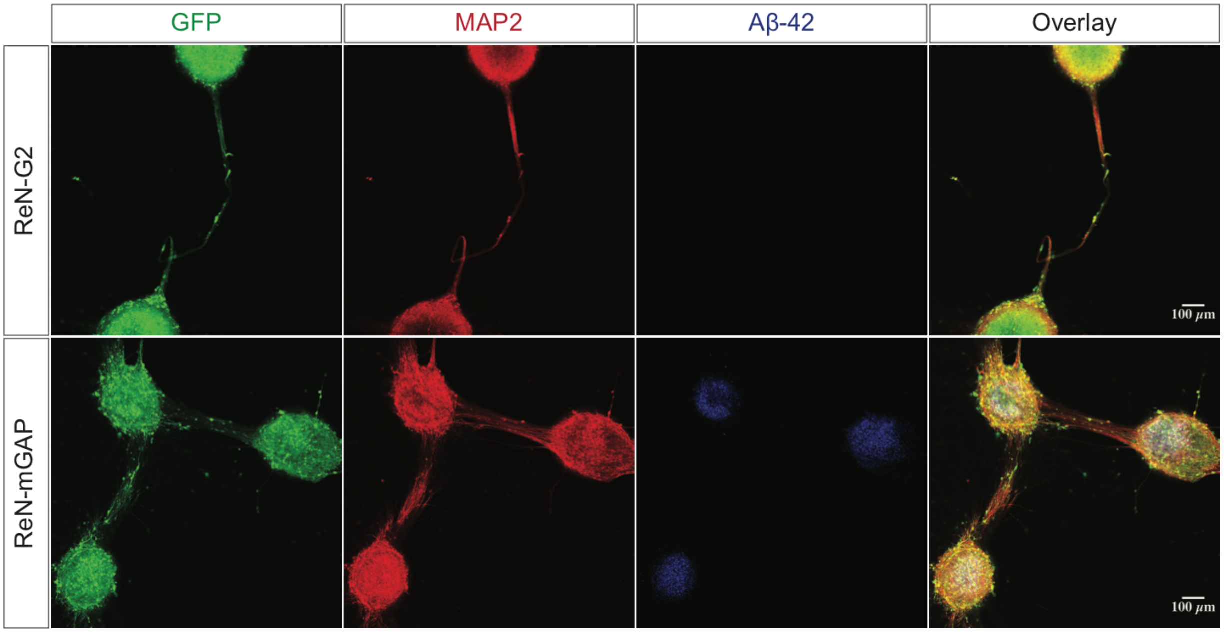

Figure S4. Three-dimensional differentiation of neurospheroids. Confocal images show neuronal extension between adjacent ReN-G2 control and ReN-mGAP FAD neurospheroids at 8-week differentiation. Increased Aβ42 deposits in FAD-derived neurospheroids (ReN-mGAP) after 8-week differentiation. Images show expression of the green fluorescent protein (GFP), microtubule-associated protein 2 (MAP-2, neuronal marker), and Aβ42 (isomer 1-42 Aβ) in the human neurospheroids.

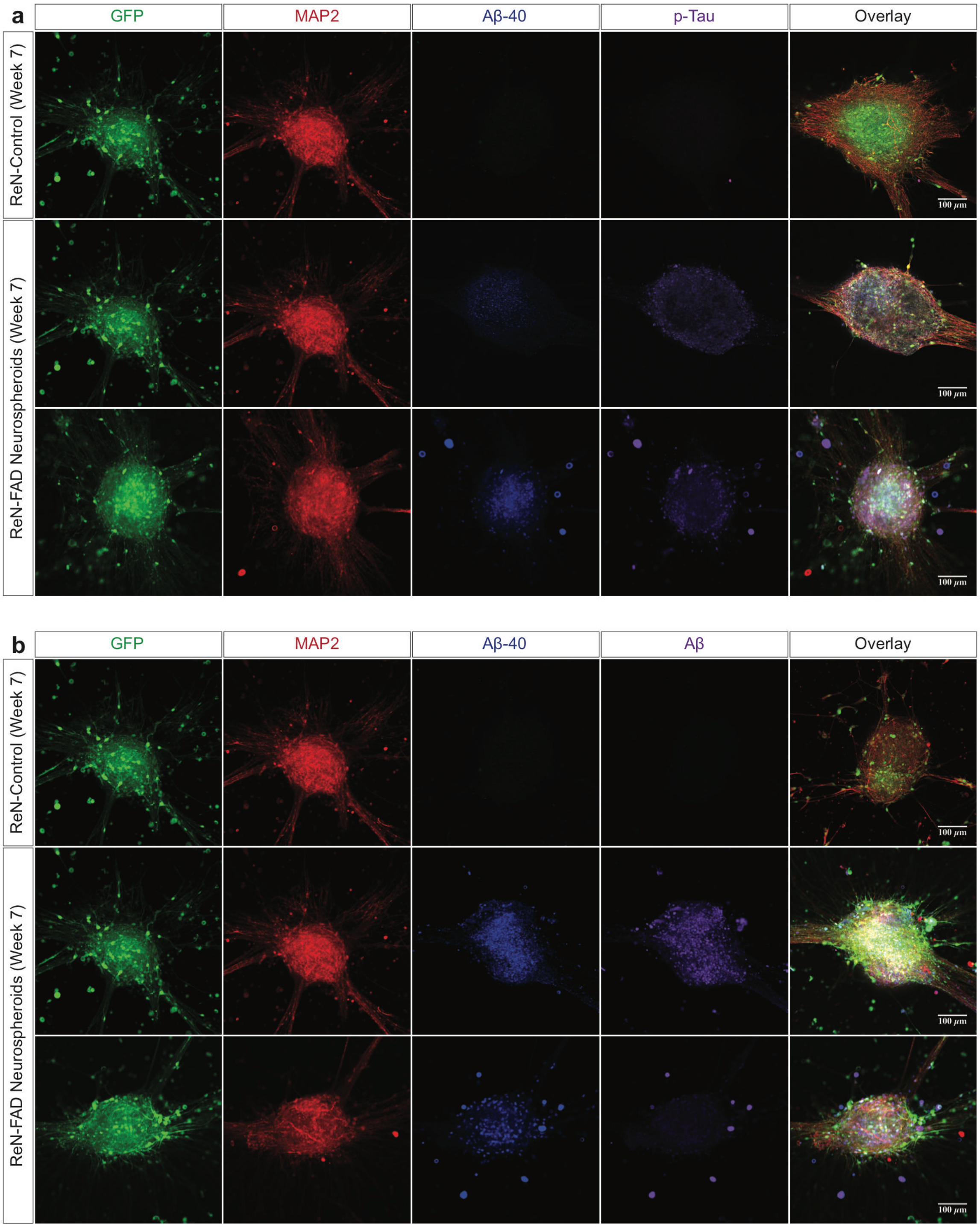

Figure S5. Recapitulating Alzheimer's disease using 3D human neurospheroids. (a) Representative confocal images show increased Aβ40 deposits and p-tau in the FAD-derived neurospheroids (ReN-mGAP) after 8-week differentiation. (b) Representative confocal images show increased Aβ40 deposits and Aβ in FAD-derived neurospheroids (ReN-mGAP) at 8-week differentiation. Images show expression of the green fluorescent protein (GFP), microtubule-associated protein 2 (MAP-2, neuronal marker), Aβ40 (isomer 1-40 Aβ), Aβ (isomers 1-40 and 1-42 Aβ), and p-tau (phospho-Tau marker) in the ReN-derived human neurospheroids.

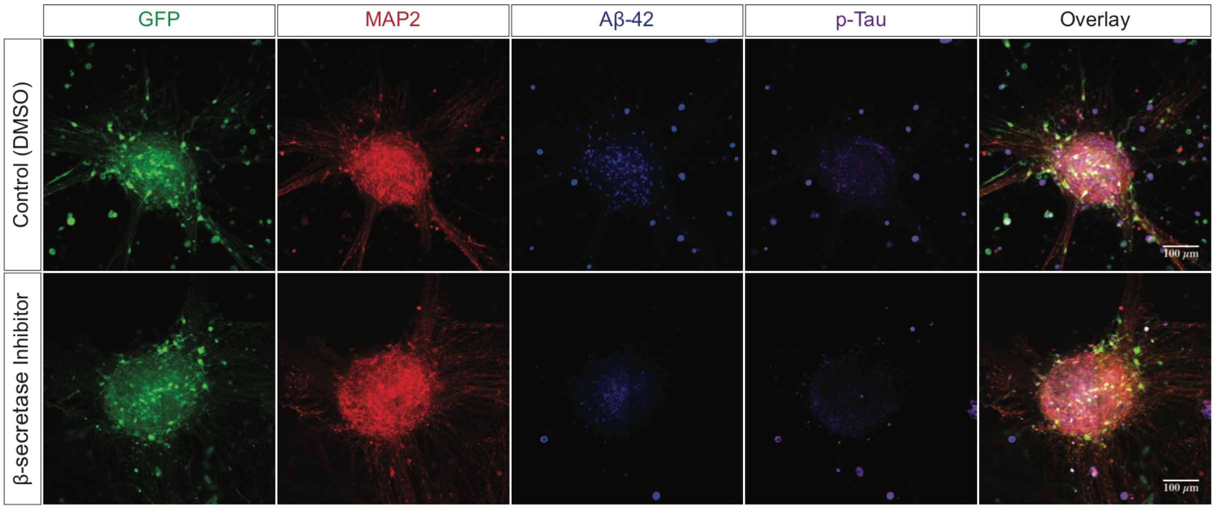

Figure S6. Qualitative evaluation of changes in amyloid-β and phosphorylated tau in 3D ReN-derived neurospheroids. Confocal images show the expression of Aβ42 (isomer 1-42 Aβ), and p-tau (phospho-Tau marker) in the FAD ReN-derived human neurospheroids (ReN-mGAP) after treatment with 1 μM β–secretase inhibitor. Images show expression of the green fluorescent protein (GFP), microtubule-associated protein 2 (MAP-2, neuronal marker), Aβ42 (isomer 1-42 Aβ), and p-tau (phospho-Tau marker) in the ReN-derived human neurospheroids.

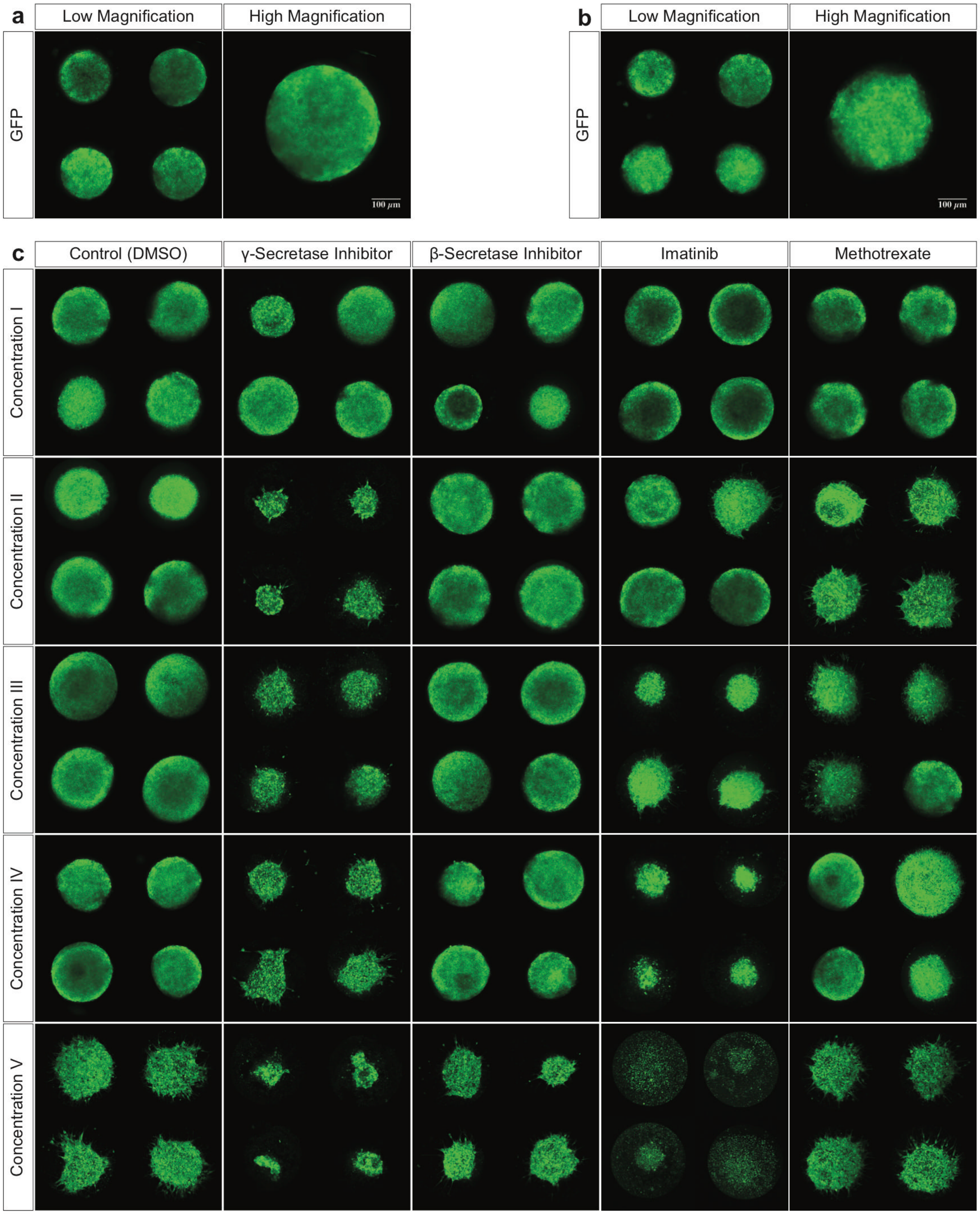

Figure S7. Qualitative evaluation of the neurospheroids in 3D platform under the effect of compounds. (a) Representative confocal images show neurospheroids without treatment at day one, and (b) at day seven after treatment. (c) Representative confocal images show qualitative comparison of the neurospheroids size and morphology at day seven after treatment with DMSO (control), γ–secretase inhibitor (Compound E), β–secretase inhibitor (LY2886721), Methotrexate, Imatinib treatments at five different concentrations.

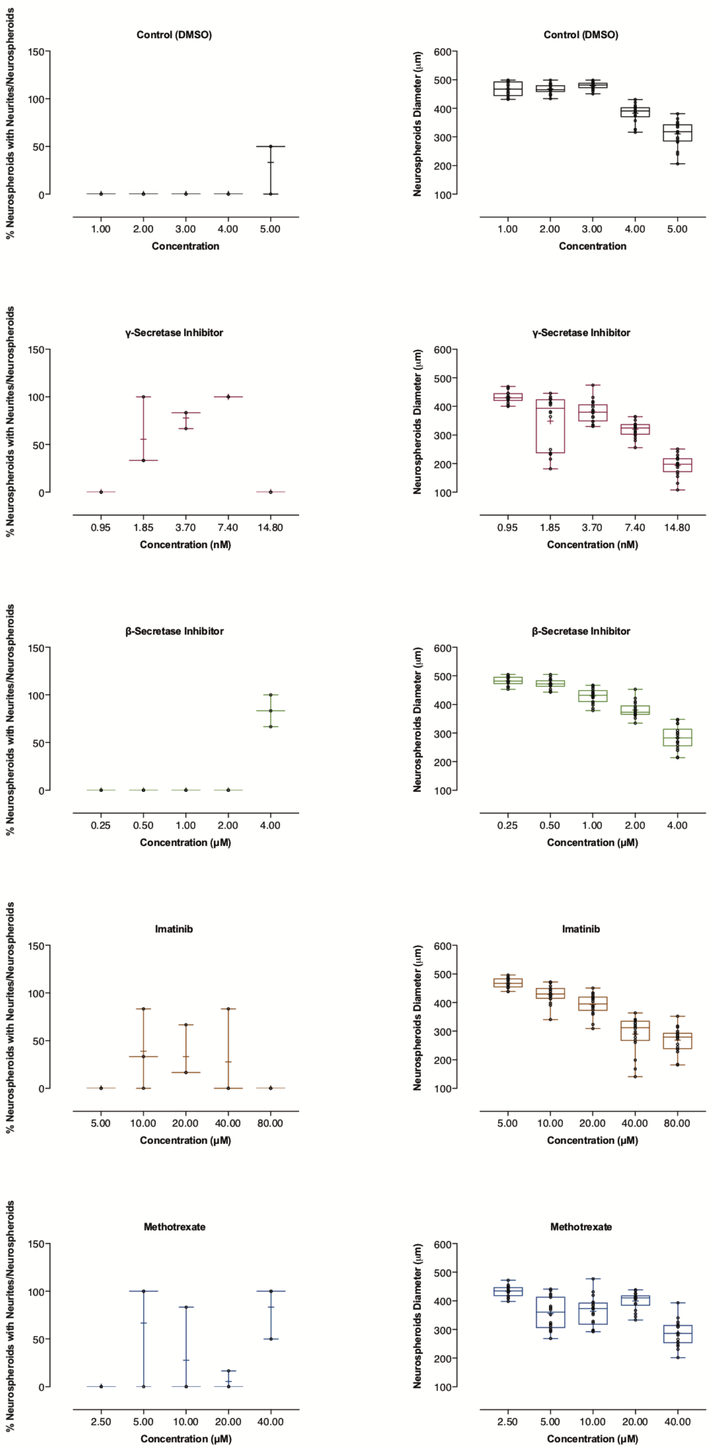

Figure S8. Quantification of the effect of γ–secretase inhibitor, β–secretase inhibitor, Methotrexate, and Imatinib treatments on neurospheroids. Graphs show the effect of various concentrations of DMSO (control), γ–secretase inhibitor (Compound E), β–secretase inhibitor (LY2886721), Methotrexate, Imatinib on neurospheroids neurites and diameter at day 7 after treatment. *$P < 0.05$; **$P < 0.01$; ***$P < 0.001$; ****$P < 0.0001$; ANOVA followed by a post hoc Dunnett's test; means ± SEM; n = 6 per each sample.

Movie S1. A Z-projection of a representative ReN-G2 derived neurospheroid (control) after 21 days of differentiation. The movie shows expression of the green fluorescent protein (GFP) in the human neurospheroid.

Movie S2. A Z-projection of a representative ReN-mGAP derived neurospheroid (FAD) after 21 days of differentiation. The movie shows the expression of green fluorescent protein (GFP) in the human neurospheroid.
